# Strong Binding of Platelet Integrin αIIbβ3 to Fibrin Clots: Potential Target to Destabilize Thrombi

**DOI:** 10.1038/s41598-017-12615-w

**Published:** 2017-10-11

**Authors:** Peter Höök, Rustem I. Litvinov, Oleg V. Kim, Shixin Xu, Zhiliang Xu, Joel S. Bennett, Mark S. Alber, John W. Weisel

**Affiliations:** 1 Department of Applied and Computational Mathematics and Statistics, University of Notre Dame, Notre Dame, IN USA; 2 Department of Cell and Developmental Biology, University of Pennsylvania School of Medicine, Philadelphia, PA USA; 3 Institute of Fundamental Medicine and Biology, Kazan Federal University, Kazan, Russia; 4 Department of Mathematics, University of California, Riverside, Riverside, CA USA; 5 Department of Medicine, Hematology-Oncology Division, University of Pennsylvania School of Medicine, Philadelphia, PA USA; 6 Department of Medicine, Indiana University School of Medicine, Indianapolis, IN USA

## Abstract

The formation of platelet thrombi is determined by the integrin αIIbβ3-mediated interactions of platelets with fibrinogen and fibrin. Blood clotting *in vivo* is catalyzed by thrombin, which simultaneously induces fibrinogen binding to αIIbβ3 and converts fibrinogen to fibrin. Thus, after a short time, thrombus formation is governed by αIIbβ3 binding to fibrin fibers. Surprisingly, there is little understanding of αIIbβ3 interaction with fibrin polymers. Here we used an optical trap-based system to measure the binding of single αIIbβ3 molecules to polymeric fibrin and compare it to αIIbβ3 binding to monomeric fibrin and fibrinogen. Like αIIbβ3 binding to fibrinogen and monomeric fibrin, we found that αIIbβ3 binding to polymeric fibrin can be segregated into two binding regimes, one with weaker rupture forces of 30–60 pN and a second with stronger rupture forces >60 pN that peaked at 70–80 pN. However, we found that the mechanical stability of the bimolecular αIIbβ3-ligand complexes had the following order: fibrin polymer > fibrin monomer > fibrinogen. These quantitative differences reflect the distinct specificity and underlying molecular mechanisms of αIIbβ3-mediated reactions, implying that targeting platelet interactions with fibrin could increase the therapeutic indices of antithrombotic agents by focusing on the destabilization of thrombi rather than the prevention of platelet aggregation.

## Introduction

**Figure 1:**
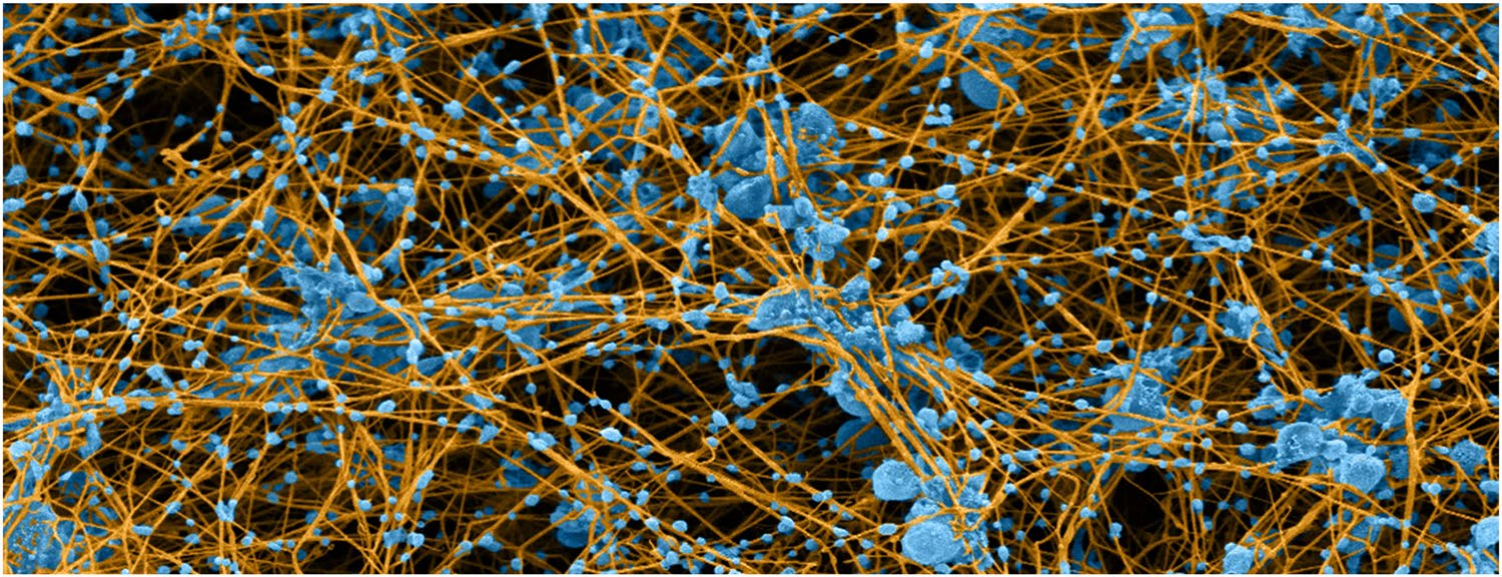
Colorized scanning electron micrograph of blood clot made by re-calcifying platelet-rich plasma. Platelets and microvesicles are colored blue and fibrin is tan. The micrograph illustrates the specific and intimate interactions between platelets and fibrin in blood clots and demonstrates that most fibrin fibers originate from platelet aggregates, where much of the thrombin is generated, while platelet-derived microvesicles decorate the fibrin fibers.

The formation of hemostatic plugs and obstructive thrombi are determined to a large extent by the integrin αIIbβ3-mediated interactions of platelets with fibrinogen and fibrin. The most studied of these interactions, fibrinogen binding to αIIbβ3, initiates platelet aggregation^1^. However, blood clotting *in vivo* is catalyzed by thrombin, which simultaneously induces fibrinogen binding to αIIbβ3 and converts fibrinogen to fibrin. Thus, after a short time period, the ligand for activated platelet αIIbβ3 binding is polymerized fibrin (p-fibrin) rather than fibrinogen or monomeric fibrin (m-fibrin)^2–5^. This is illustrated by the scanning electron micrograph of a blood clot formed by re-calcifying platelet-rich plasma shown in Fig. 1, in which fibrin strands emanate from platelet aggregates. This platelet-fibrin meshwork enables platelet-mediated clot retraction and has important functional consequences such as compression of clot-enmeshed erythrocytes causing a dramatic decrease of clot permeability^6^ and clot shrinkage enabling blood flow past otherwise obstructive thrombi^7^. Surprisingly, there is little understanding of αIIbβ3 interaction with fibrin polymers^8,9^. Previously, we used an optical trap-based system to measure the binding of single αIIbβ3 molecules to fibrinogen and m-fibrin^10–13^. Here, we extended these studies to αIIbβ3 binding to p-fibrin, finding that αIIbβ3 has a higher probability of forming mechanically stable interactions with p-fibrin than with fibrinogen. Targeting platelet binding to p-fibrin, rather than platelet aggregation, may be a way to increase the therapeutic indices of anti-platelet agents.

## Results and Discussion

### Model system to study interactions of polymerized fibrin with αIIbβ3

**Figure 2:**
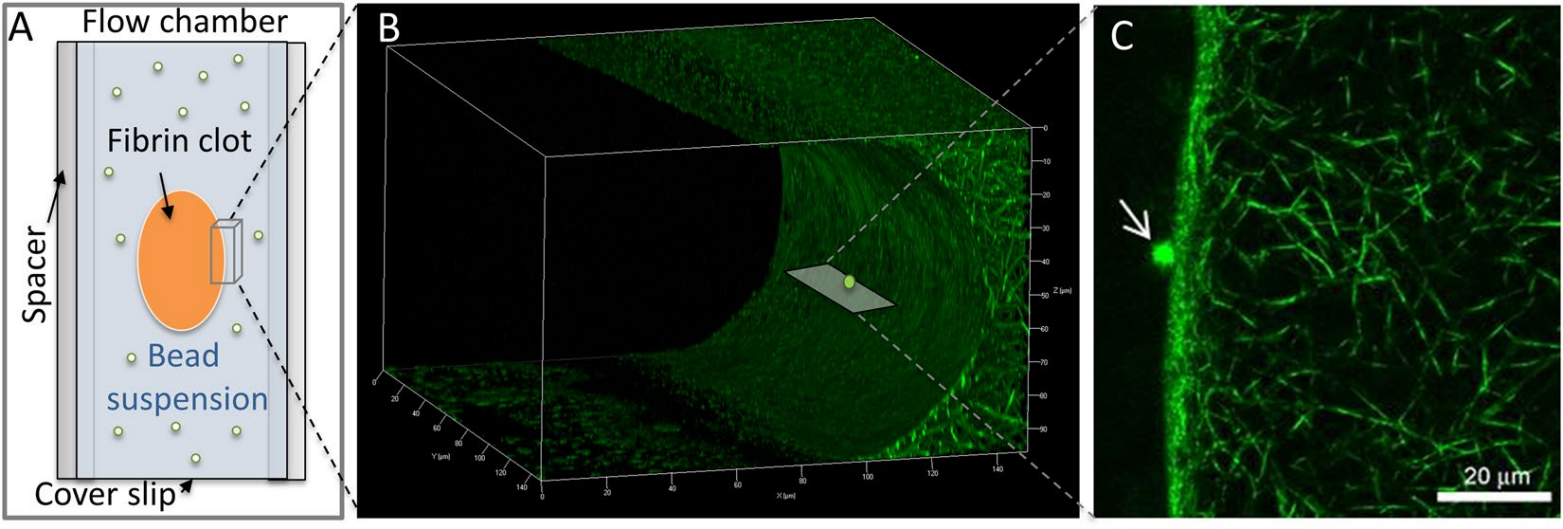
(**A**) Schematic representation of the fibrin clot formed as a strip inside a flow chamber, providing a natural hydrated interface for binding of the integrin αIIbβ3. The clot is surrounded by a suspension of microscopic αIIbβ3-coated latex beads, one of which is trapped by the focused laser beam and oscillated to touch repeatedly the edge of the fibrin clot (see Methods). When the bead interacts with fibrin, the tension is produced and displayed as a force signal that is proportional to the strength of αIIbβ3-fibrin binding. (**B**) Z-stacked confocal microscopy images of the flow chamber and a lateral section of a fibrin clot spanning 100 µm of sample thickness. The clot was made from re-calcified plasma supplemented with Alexa-Fluor 488-labeled human fibrinogen. The compressed appearance of the clot results from the preparation of the flow chamber, such that a coverslip is placed and pressed down gently on top of the plasma strip to prevent clot dehydration and to create a cover for the chamber. Because the top and bottom of the clot are extended laterally by slight compression, all optical trap experiments were conducted with a trapped bead making contacts with the planar surface of the clot in the center of the chamber, where there is no compression as shown in **B**. (**C**) Top view. A latex bead (arrow) is shown making interactions with the surface edge of the fibrin clot.

Quantitative measurements of platelet binding to insoluble p-fibrin are difficult methodologically and conventional experimental approaches have been ineffective. Previously, we used optical trap-based force spectroscopy to determine the nanomechanics and two-dimensional kinetics of the interaction of αIIbβ3 with fibrinogen, m-fibrin, and the fibrinogen-related peptides cRGDFK, and γC-12^11–17^. However, to measure the interaction of αIIbβ3 with polymeric rather than monomeric proteins, it was necessary to re-configure our optical trap system. In the re-configured system, a fibrin clot was formed as a narrow strip in the center of a flow chamber (Fig. 2A), providing a hydrated interface for αIIbβ3 binding. Imaging the clots by confocal microscopy revealed a uniform network of high fiber density with well-defined edges allowing for precise visual control of contact with αIIbβ3-coated beads. The compressed appearance of the upper surface of the clot (Fig. 2B) resulted from preparation of the flow chamber in which a coverslip is gently pressed on top of the fibrin strip to prevent clot dehydration and to create a cover for the chamber. Because the top and bottom of the clot are extended laterally by slight compression, all optical trap experiments were conducted with a laser-trapped αIIbβ3-coated bead making repeated contacts with the planar surface of the clot at the center of the chamber where there is no compression (Fig. 2C). Rupture force signals generated during repeated contacts were then collected into 10 pN-wide bins and plotted against the average force for that bin after normalizing for the total number of interaction cycles.

### Interactions between αIIbβ3 and polymerized fibrin

**Figure 3:**
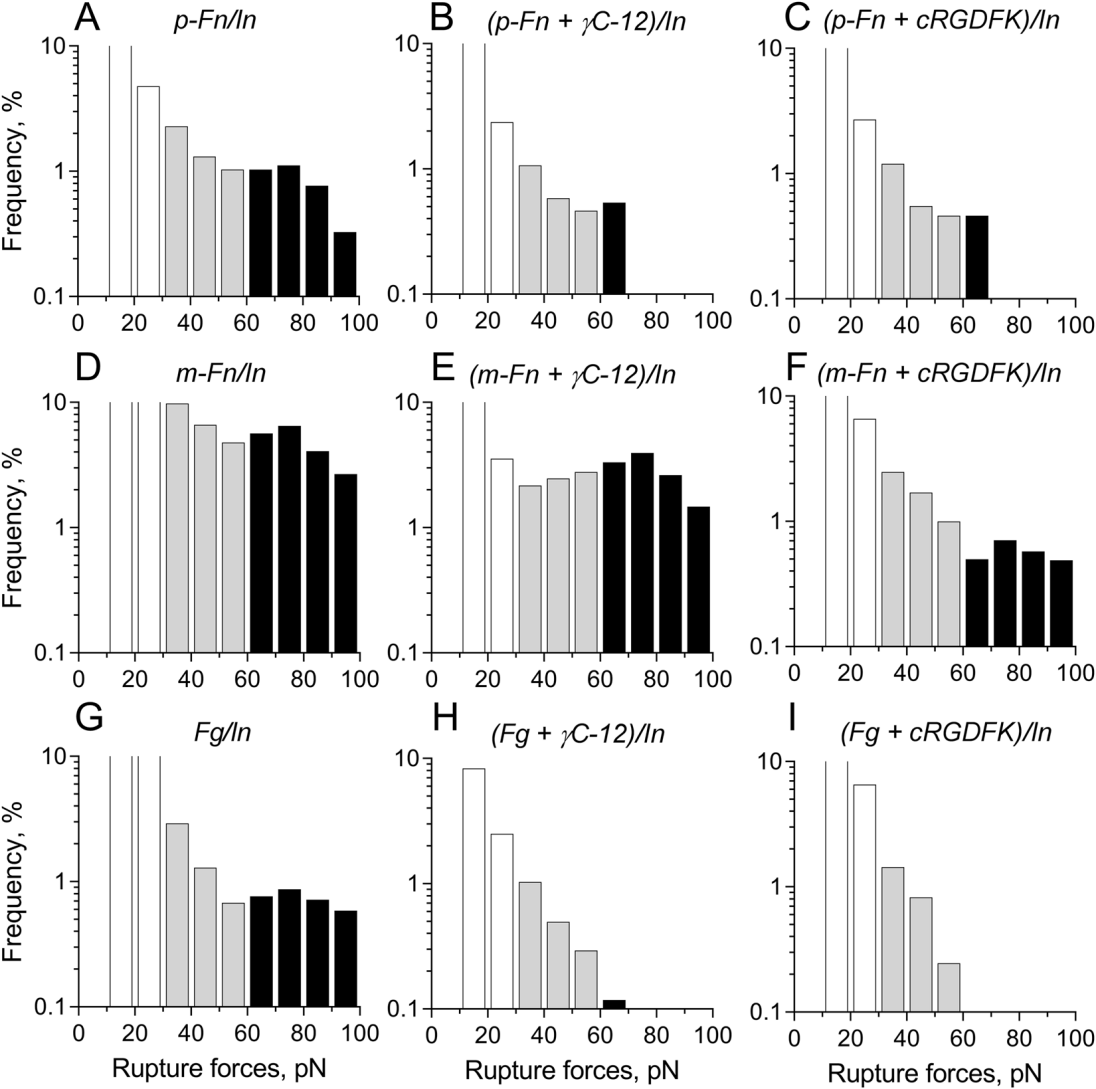
The panel of rupture force histograms in 10-pN wide bins of the interactions between Mn^2+^-activated αIIbβ3 integrin and polymerized fibrin (**A–C**), αIIbβ3 and monomeric fibrin (**D–F**), αIIbβ3 and fibrinogen (**G–I**) in the absence (**A**,**D**,**G**) and presence of an αIIbβ3 antagonist. Two competitive antagonists were used to suppress specific αIIbβ3-fibrin interactions: dodecapeptide (γC-12) that mimics the C-terminal AGDV-containing 400–411 sequence of the fibrinogen γ chain (**B**,**E**,**H**) or the cyclic RGDFK peptide containing the Arg-Gly-Asp (RGD) integrin-binding motif that is present as residues 95–97 and 572–574 of the fibrin(ogen) (A)α chains (**C**,**F**,**I**). The rupture force profiles are segregated into three regimes, namely low forces <30 pN (shown as blank bins) that represent optical artifacts and/or non-specific background surface-to-surface interactions; moderate forces 30–60 pN (grey bins) representing the lower affinity αIIbβ3-fibrin(ogen) complexes, partially overlapped with the non-specific interactions; and high forces >60 pN (dark bins) representing higher affinity αIIbβ3-fibrin(ogen) complexes. The moderate forces are incompletely abrogated by the antagonists, while the strong forces are almost fully suppressed, corresponding to partial and full specificity, respectively. The total number of contacts is n = 9,685 for A, n = 5,871 for B, n = 8,967 for C, n = 11,344 for D, n = 8,937 for E, n = 9,633 for F, n = 10,386 for G, n = 7,926 for H, n = 6,937 for I.

To validate our re-configured optical trap-based system, we measured rupture forces between movable latex beads coated with αIIbβ3 and silica pedestals coated irreversibly with either fibrinogen or m-fibrin. As we found previously, αIIbβ3-fibrinogen (Fig. 3G) and αIIbβ3-m-fibrin (Fig. 3D) interactions had a characteristic bimodal rupture force distribution with rupture forces of up to 140 pN^13^. The rupture force distributions consisted of the sum of exponentially decreasing weak to moderate (20–60 pN) forces, and a Gaussian-like distribution of strong (>60 pN) rupture forces peaking at 70–80 pN and were specific for αIIbβ3 binding because they were competitively inhibited by the free fibrinogen γC-dodecapeptide (γC-12) and by cRGDFK (Table 1, Fig. 3E,F,H,I). Because the pedestals were saturated with fibrinogen and m-fibrin, the probability of specific αIIbβ3 binding was determined primarily by the intrinsic reactivity of these ligands towards αIIbβ3. However, as we found previously, αIIbβ3 binding to m-fibrin-coated beads was only moderately sensitive to competitive inhibition by the γC-12 and cRGDFK, implying that αIIbβ3 binding to m-fibrin could be mediated by binding sites other than the C-termini of the γ chains or either of the two α chain RGD motifs. Taken together, these rupture force measurements confirm that despite reversing the location of receptor and ligand, the re-configured system faithfully reproduces our previous results.

**Table 1:** Cumulative probability (M ± SD) of interactions between αIIbβ3 and polymerized fibrin (p-Fn), monomeric fibrin (m-Fn) and fibrinogen (Fg).

Interacting molecules and conditions	Cumulative binding probability (%)	
	30–60 pN	>60 pN
p-Fn/Mn^2+^-activated αIIbβ3	4.6 ± 0.9	3.2 ± 0.7
p-Fn/Mn^2+^-activated αIIbβ3 + γC-12	2.1 ± 0.5^*^	0.8 ± 0.2^**^
p-Fn/Mn^2+^-activated αIIbβ3 + cRGDFK	2.2 ± 0.7^*^	0.6 ± 0.2^**^
p-Fn/Mn^2+^-activated αIIbβ3 + EDTA	2.2 ± 0.8^*^	0.10 ± 0.06^***^
p-Fn/Mn^2+^-activated αIIbβ3 + eptifibatide	2.4 ± 0.7^*^	0.5 ± 0.2^**^
p-Fn/Non-activated αIIbβ3	4.7 ± 1.1	1.7 ± 0.5^*^
p-Fn/BSA	1.4 ± 0.4^**^	0.2 ± 0.1^***^
m-Fn/Mn^2+^-activated αIIbβ3	21 ± 2	22 ± 2
m-Fn/Mn^2+^-activated αIIbβ3 + γC-12	7.4 ± 2.1^***^	12.3 ± 3.2^***^
m-Fn/Mn^2+^-activated αIIbβ3 + cRGDFK	5.1 ± 1.5^***^	2.7 ± 1.2^***^
Fg/Mn^2+^-activated αIIbβ3	4.8 ± 0.8	3.6 ± 0.6
Fg/Mn^2+^-activated αIIbβ3 + γC-12	1.8 ± 0.6^**^	0.2 ± 0.1^***^
Fg/Mn^2+^-activated αIIbβ3 + cRGDFK	2.5 ± 0.3^*^	0.3 ± 0.1^***^

*p < 0.05, **p < 0.01, ***p < 0.001 compared to Mn^2+^-activated integrin, unpaired two-tail *t*-test.

Next, we asked whether the interaction between beads covalently-coated with Mn^2+^-activated αIIbβ3 and an immobilized fibrin network produces measurable rupture forces and whether the rupture forces are similar to those generated when αIIbβ3 binds to immobilized m-fibrin^13,15,16^. As shown in Fig. 3A, the rupture force distribution of αIIbβ3 bound to p-fibrin displayed two distinct regimes, one with weaker rupture forces of 30–60 pN and a second with stronger rupture forces >60 pN that peaked at 70–80 pN. These rupture force regimes are very similar to the force profiles resulting from αIIbβ3 bound to m-fibrin^13^, the major difference being a significantly higher cumulative binding probability for αIIbβ3 binding to m-fibrin throughout the entire force range likely due to either the increased density of immobilized m-fibrin and/or an enhanced on-rate for αIIbβ3 binding to m-fibrin versus p-fibrin (Table 1).

To insure that the rupture forces we measured resulted from specific αIIbβ3-p-fibrin interactions, we performed two sets of control experiments. First, we either inactivated αIIbβ3 using EDTA^1,18^ or replaced it with the irrelevant protein bovine serum albumin (BSA). While inactivating αIIbβ3 using EDTA or replacing it with BSA partially decreased rupture forces in the weaker force regime, rupture forces >60 pN were abrogated under both sets of conditions (Table 1). Second, we inhibited αIIbβ3 binding to p-fibrin using eptifibatide, cRGDFK, and γC-12^13,15^. Each peptide decreased the cumulative binding probability in the weaker rupture force regime by ~50%, whereas their inhibitory effect on the stronger rupture forces was significantly greater (Table 1, Fig. 3B and C). Thus, the results suggest that a least a portion of the weaker rupture forces resulted from non-specific protein-protein interactions, whereas the stronger rupture forces results almost exclusively from the specific binding αIIbβ3 to p-fibrin. In support of this conclusion, we found that the probability of rupture forces >60 pN was significantly greater (p < 0.05) when experiments were performed using Mn^2+^-activated αIIbβ3 rather than the non-activated integrin (Table 1). It is noteworthy that purified αIIbβ3 consists of a mixture of low- and high-affinity forms, both of which are sensitive to the inhibitory effects of the antagonists, albeit with a different degree of susceptibility. Resting platelets adhere weakly to fibrinogen-coated surfaces, implying that αIIbβ3 is residing on the unstimulated cells in a low-affinity form that is still capable of interacting with fibrinogen and fibrin.

### Kinetic model and parameters of the interaction of αIIbβ3 with polymerized fibrin

The bimodal distribution of rupture forces implies that αIIbβ3 interacts with the p-fibrin through a two-step mechanism similar to the way it interacts with fibrinogen^19,20^. In the latter case, αIIbβ3 exists in two interconvertible states, a lower affinity state *R*_1_ and a higher affinity state *R*_2_, corresponding to lower (*LR*_1_) and higher affinity (*LR*_2_) αIIbβ3-fibrinogen complexes. Further, it suggests that our two-state kinetic model for αIIbβ3 binding to fibrinogen^19^ can be applied to describe the dynamics of αIIbβ3 binding to p-fibrin as follows:

1 $$\begin{array}{c}L{R}_{1}\mathop{\to }\limits^{\,{k}_{1}\,}\,L\,+\,R\\ {r}_{12}\downarrow \uparrow {r}_{21}\\ L{R}_{2}\mathop{\to }\limits^{\,{k}_{2}\,}\,L\,+\,R\end{array}$$

where *k*_1_ is the forced unbinding rate of the lower affinity complexes and is approximated by the Bell model: ${k}_{1}={k}_{10}\,\exp (\frac{f{x}_{1}}{{K}_{B}T})$ with *k*_10_ being the force-free unbinding rate and *x*_1_ being the critical elongation (or transition state distance) of the bond *LR*_1_. *k*_2_ is the forced unbinding rate of the higher affinity complexes and is approximated by ${k}_{2}={k}_{20}\,\exp (\frac{f{x}_{2}}{{K}_{B}T}){\rm{m}},$ with *k*_20_ being the force-free unbinding rate and *x*_2_ being the critical transition state distance of the *LR*_2_ bond. *r*_12_ and *r*_21_ are the transition rates from *LR*_1_ to *LR*_2_ and vice versa. By letting *P*_1_ and *P*_2_ be the survival probabilities of states *LR*_1_ and *L*, the dynamics of the probabilities can be calculated by the following system^19^,

2 $$\frac{d{P}_{1}}{dt}=-({r}_{12}+{k}_{1}){P}_{1}+{r}_{21}{P}_{2},$$

3 $$\frac{d{P}_{2}}{dt}=-({r}_{21}+{k}_{2}){P}_{2}+{r}_{12}{P}_{1}.$$

If we assume that the interconversion between the states is fast, i.e. ${r}_{12}{P}_{1}={r}_{21}{P}_{2}$ and defines $\,P={P}_{1}+{P}_{2}$, then the above equations can be simplified as in^20^

4 $$\frac{dP(t)}{dt}=-\frac{({k}_{1}{{\rm{\Psi }}}_{0}+{k}_{2}\,\exp (\frac{f{y}_{12}}{{K}_{B}T}))P(t)}{{{\rm{\Psi }}}_{0}+\exp (\frac{f{y}_{12}}{{K}_{BT}})},$$

5 $$P(0)=1,$$

where the parameter ${{\rm{\Psi }}}_{0}=\frac{{P}_{1,0}}{{P}_{2,0}}=\frac{{r}_{21}}{{r}_{12}}$ is the force-free equilibrium constant, and *y*_12_ is the distance between the energy wells *LR*_1_ and *LR*_2_. The initial probabilities of the two states are represented by ${{\rm{\Psi }}}_{0}$ as ${P}_{10}=\frac{{\Psi }_{0}}{1+{\Psi }_{0}},\,{P}_{20}=\frac{1}{1+{\Psi }_{0}}\,$ ^19^.

**Figure 4:**
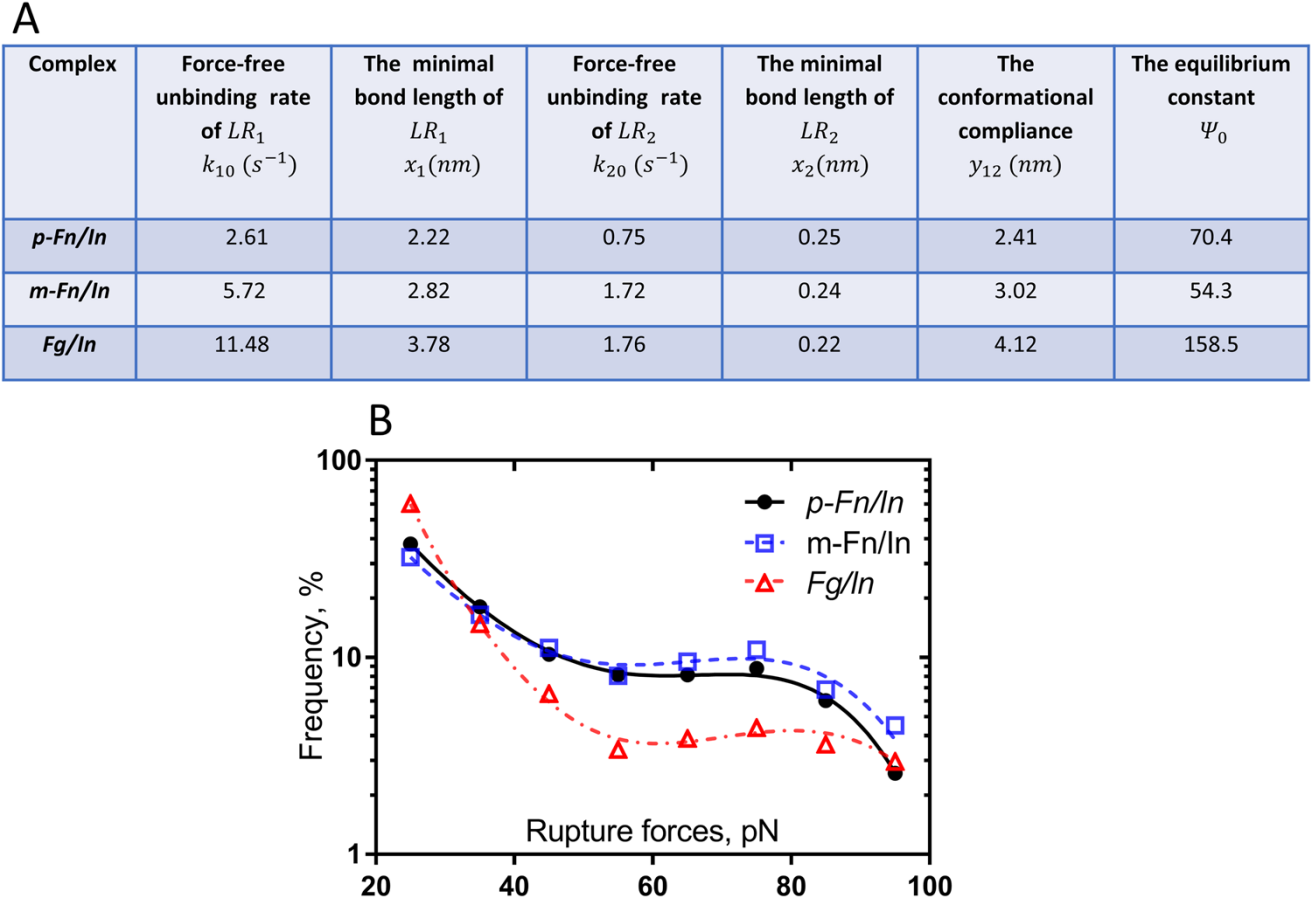
(**A**) Analytic approximation parameters for the interactions between Mn^2+^-activated αIIbβ3 integrin (In) and polymeric fibrin (p-Fn), monomeric fibrin (m-Fn) and fibrinogen (Fg), respectively. (**B**) Experimental rupture force profiles (symbols) of the interactions of αIIbβ3 with polymeric fibrin (p-Fn), monomeric fibrin (m-Fn), and fibrinogen (Fg) fitted to the Bell function (see Supplementary Information). Signals that appeared as forces below 20 pN, corresponding to optical artifacts and non-specific binding events were excluded from the analysis. The total number of contacts is n = 5,871 for p-Fn, n = 11,344 for m-Fn, and n = 10,386 for Fg. The numerical parameters extracted from the fitting analysis are presented in (**A**).

The results of fitting the normalized experimental binding frequency distributions under rupture forces in the range 20–100 pN using Eq. (4) are shown in Fig. 4, confirming that the two-state model yields a good approximation of the experimental data.

The calculated unbinding rates and transition state distances are summarized in Fig. 4A. The experimental and modeled results point to notable differences in the affinity by which αIIbβ3 binds to fibrinogen vs p-fibrin. First, the force free unbinding rate of the low-affinity αIIbβ3-fibrinogen complex is 2–4 times greater than that of αIIbβ3 and p-fibrin. This could explain the relatively high frequency of αIIbβ3-fibrinogen dissociation at low rupture forces and the exponential decrease in unbinding under weak to moderate (20–60 pN) forces (Fig. 3G). Second, from the definition of the equilibrium constant, it follows that the initial probabilities of high-affinity state of αIIbβ3-fibrin interactions (0.0140 for p-fibrin, and 0.0181 for m-fibrin) are 2-3x larger than αIIbβ3-fibrinogen (0.0063). This could explain why αIIbβ3-fibrin is more stable under high rupture force (>60 pN). Further, the lower force-free unbinding rates in αIIbβ3-p-fibrin interactions compared with those between αIIbβ3 and m-fibrin suggests that a more stable interaction with the αIIbβ3 is attained after fibrin polymerizes.

To summarize, we found that like αIIbβ3 binding to fibrinogen and m-fibrin, αIIbβ3 binding to p-fibrin can be segregated into two binding regimes that reflect lower- and higher-affinity activation states of αIIbβ3. Although the higher maximal rupture forces of αIIbβ3 bound to fibrinogen and m-fibrin suggest that αIIbβ3 binding to these ligands is stronger than binding to p-fibrin, the smaller forces in the p-fibrin histograms are likely due to the formation of multiple αIIbβ3-fibrin bonds with higher binding forces (140 pN) beyond the maximal power of the optical trap. Similarly, the lower binding probability reflects the formation of multiple αIIbβ3 bonds that were unmeasurable and missing in the registered rupture force spectra. This presumption is supported by the computed mechanical stability of the bimolecular αIIbβ3-ligand complexes, which has the following order: fibrin polymer > fibrin monomer > fibrinogen. These quantitative differences reflect the distinct specificity and underlying molecular mechanisms of αIIbβ3-mediated reactions, implying that targeting platelet interactions with fibrin could increase the therapeutic indices of antithrombotic agents by focusing on destabilization of developing or formed thrombi rather than prevention of platelet aggregation. We recently demonstrated differences in the binding specificity between αIIbβ3-Fg and αIIbβ3-m-Fn interactions^13^. We found that the number of integrin-binding sites in m-fibrin is not limited to its RGD and γC-12 motifs and that at least three potential integrin-binding motifs that become exposed upon Fg to Fn conversion and are potential targets to specifically inhibit the interaction of activated platelets with p-Fn.

## Materials and Methods

### Optical trap-based force spectroscopy

Our model system designed to measure the nanomechanics of bimolecular interactions is based on a custom-built optical trap previously described in detail^15^ with some modifications. This system permits studies of single-molecule force-induced unbinding between two surfaces coated with interacting proteins; one surface is motionless and the other consists of a laser-trapped oscillating microsphere (bead). The core of the laser trap system is a Zeiss Axio Observer A1 inverted microscope and a 60 × 1.3 numerical aperture Fluor lens combined with an FCBar Nd:YAG laser (λ = 1,064 nm) with 4Ws of power in continuous TEM-00 mode. A computer-operated two-dimensional acousto-optical deflector is used to control the trap position and hence the position of the trapped bead. The force exerted by the trap on the bead displaced from the laser beam focus is measured with a quadrant detector. The system enables control of the bead/trap oscillation frequency and amplitude, the magnitude of the compressive force, and the magnitude of the tensile force during dissociation of the surfaces coated with interacting molecules. All experiments are conducted at an average trap stiffness of 0.10 ± 0.02 pN/nm as computed from measurements of the bandwidth of Brownian motion for different beads^21^. Custom-written software on the LabVIEW^®^ platform (National Instruments, Austin, TX) is used to control and record laser beam deflection, move the piezoelectric stage (Queensgate, Birkshire, UK), and for subsequent off-line data analysis.

### Measurement of the interaction of αIIbβ3 with fibrinogen or fibrin at the single-molecule level

We measured the interaction of single αIIbβ3 molecules with fibrinogen or fibrin monomer using a modification of our optical trap-based force spectroscopy system^11–17^ in which purified αIIbβ3 was immobilized on freely-moving latex beads, while fibrinogen or monomeric fibrin was bound to silica beads attached to the bottom of a fluid-filled flow chamber. Under visual microscopic control, a bead coated with αIIbβ3 was trapped by a focused laser beam and moved in an oscillatory manner so that it tapped a stationary fibrinogen- or fibrin monomer-coated pedestal (Fig. 1). When the immobilized αIIbβ3 on the bead interacted with fibrinogen or fibrin monomer on the pedestal, tension was produced when the bead was displaced from the laser focus until the αIIbβ3-ligand bond ruptured. The applied force was then displayed as a signal proportional to the strength of αIIbβ3-ligand binding. Such signals were on the order of picoNewtons and quantitatively characterize the interactions of αIIbβ3 with fibrinogen or fibrin monomer at the nanomechanical molecular level. The key modification that enabled us to measure the interaction of αIIbβ3 with polymeric fibrin was that the motionless surface was not a fibrinogen or fibrin-coated pedestal, but a fibrin clot formed inside a flow chamber. Then one freely moving αIIbβ3-coated bead was trapped by a focused laser beam, brought close to the fibrin clot, and oscillated with a triangular waveform in order to repeatedly touch the edge of the clot, mimicking the physical interaction of an activated platelet with a fibrin network.

### Coating latex beads with αIIbβ3

Purified human αIIbβ3 (Abcam, Cambridge, MA) at 1 mg/ml in 20 mM HEPES, pH 7.4, containing 150 mM NaCl, 1 mM CaCl_2_ and 30 mM 30 mM n-octyl-β-D-glucoside, was activated with 1 mM MnCl_2_ for 30 min at 37 °C. The activated αIIbβ3 was covalently attached to 2.0 µm in diameter carboxylate-modified latex beads (Bangs Laboratories, Fishers, IN) using N-(3-dimethylaminopropyl)-N′-ethylcarbodiimide hydrochloride as a crosslinking agent in a two-step procedure. The beads were activated by mixing 1 ml of the 0.1 M 2-(N-morpholino)ethanesulphonic acid, pH 5.2, 20 µl of a 10% bead suspension and 3 mg of dry carbodiimide, followed by constant shaking for 15 min at room temperature. The activated beads were sedimented at 4,000 g for 2 min, washed once with binding buffer (0.055 M borate buffer, pH 8.5), sedimented again, and re-suspended in 1 ml of 50 µg/ml Mn^2+^-activated αIIbβ3 in the binding buffer. After a 30 min incubation with stirring at room temperature, the beads were sedimented and resuspended in 1 ml of bovine serum albumin (2 mg/ml in 0.055 M borate buffer, pH 8.5) to block the remaining active groups. Following this protocol, the surface density of functional αIIbβ3 molecules capable of binding ^125^I-fibrinogen was found to be 3,072 ± 412 molecules/µm^2^. αIIbβ3-coated beads were freshly prepared and mildly sonicated to disrupt aggregates before each experiment.

### Coating silica pedestals with fibrinogen and monomeric fibrin

Purified human fibrinogen (Hyphen BioMed, France) was bound covalently to 5-µm spherical silica pedestals anchored to the bottom of a chamber as previously described^11,13,15^. Briefly, pedestals coated with a thin layer of polyacrylamide were activated with 10% glutaraldehyde (30 min, 37 °C), after which fibrinogen (1 mg/ml in the activation mixture) was immobilized for 2 hr at 4 °C. The chamber was then washed with 20 volumes of the same buffer to remove non-covalently adsorbed protein, blocked with 2 mg/ml bovine serum albumin (BSA) in 0.055 M borate buffer, pH 8.5, 150 mM NaCl, 3 mM CaCl_2_ for 30 min at 4 °C, and equilibrated with 20 mM HEPES buffer, pH 7.4, containing 150 mM NaCl. To generate surfaces coated with monomeric fibrin, the fibrinogen-coated pedestals were treated with human α-thrombin (1 U/ml, 37 °C, 1 hr) followed by thorough washing (20 volumes of the flow chamber) and equilibration in 0.1 M HEPES buffer, pH 7.4, containing 150 mM NaCl, 3 mM CaCl_2_, 1 mM MnCl_2_, 2 mg/ml BSA, and 0.1% (v/v) Triton X-100. Treatment with thrombin has been previously shown to generate biologically active fibrin molecules on the bead surface^10,22^. Because the monomeric fibrin was covalently attached to the bead surface, substantial fibrin oligomerization was not possible.

### Formation of a fibrin clot inside a flow chamber

A fibrin network was formed using either purified human fibrinogen or platelet-poor plasma obtained from citrated human whole blood by centrifugation at 1,500 g for 15 minutes at room temperature. Plasma samples from more than 10 healthy donors were pooled, aliquoted, and frozen. A new aliquot was thawed at 37 °C for 30 min before use. The plasma was re-calcified with 25 mM CaCl_2_ and 10 µl was immediately applied as a strip in the center of a microscopic flow chamber (Fig. 1). After 30 min at 37 °C in a humid atmosphere, a clot with well-defined boundaries was formed. To prevent drying of the clot, assay buffer [0.1 M HEPES, pH 7.4, 2 mg/ml BSA, and 0.1% (v/v) Triton X-100] was flowed into the chamber after clot formation. The clot was then kept at 4 °C for 1–4 hours before use. To compare experimental data obtained with fibrin from plasma with fibrin made from purified fibrinogen, 1 mM CaCl_2_ and 0.5 U/ml thrombin (ERL, South Bend, IN) were added to 1 mg/ml purified fibrinogen in 20 mM HEPES buffer, pH 7.4, containing 150 mM NaCl. 10 µl of the mixture was immediately applied to an experimental flow chamber and allowed to clot. Following clot formation, the chamber was filled with the assay buffer, and used on the same day of the experiment in the same conditions as plasma clots.

### Fluorescent confocal microscopy of fibrin clots

For morphological characterization of the fibrin clot, Alexa-Fluor 488-labeled human fibrinogen (Molecular Probes, Grand Island, NY) was added to plasma at a final concentration of 0.04 mg/ml before re-calcification and clot formation. The fibrin clot was imaged using Zeiss LSM710 laser scanning confocal microscope equipped with a Plan Apo 40x water immersion objective lens (N/A = 1.2) to provide high-resolution z-stack images spanning 100 µm of sample thickness. An argon laser beam with wavelength of 488 nm was used for fluorescent and reflectance confocal microscopy of the labeled fibrin. The z-stack distance between slices was set as 0.5 µm with 1024 × 1024 pixels resolution for each slice.

### Measurement of αIIbβ3-fibrin binding strength

All measurements were conducted in an assay buffer composed of 0.1 M HEPES, pH 7.4, 2 mg/ml BSA, and 0.1% (v/v) Triton X-100. 1 µl of the αIIbβ3-coated latex bead suspension (10^7^ beads/ml) in 50 µl of assay buffer was flowed into the chamber containing a either fibrin-coated pedestals or pre-formed fibrin clot. The chamber was then placed on the microscope stage of the optical trap setup, a single αIIbβ3-coated latex bead was trapped by the focused laser beam and moved manually to within 1–2 µm of the edge of a pedestal or a fibrin network. After beginning oscillation of the bead, the distance between the bead and the interacting surface was reduced in 20-nm steps using a piezostage until they touched each other repeatedly, allowing for direct interactions between the surface-attached αIIbβ3 and either monomeric fibrin or polymerized fibrin fibers. The position of the optical trap was oscillated in a triangular waveform at 5 Hz and constant peak-to-peak amplitude of 800 nm. The tension produced when an αIIbβ3 molecule on the latex bead interacted with fibrin was sensed and displayed as a force signal that was proportional to the strength of αIIbβ3-fibrin binding.

### Data processing and analysis

Rupture force signals from several tens of αIIbβ3-coated beads interacting with fibrin were collected for each experimental condition, so the total number of touching events analyzed varied from 10^3^ to about 10^5^. Individual forces measured during each contact-detachment cycle were collected into 10-pN-wide bins and plotted against the average force for that bin after normalizing for the total number of interaction cycles. The percentage of events in a particular force range (bin) represents the frequency (probability density) of rupture events at that tension. Optical artifacts observed with or without trapped latex beads produce signals that appeared as forces below 10 pN. Accordingly, rupture forces in this range were not considered when the data were analyzed.

## Electronic supplementary material

Supplementary information
