# Supplementary material for: Strong Binding of Platelet Integrin αIIbβ3 to Fibrin Clots: Potential Target to Destabilize Thrombi
Source: Sci Rep. 2017 Oct 11;7:13001. doi: 10.1038/s41598-017-12615-w (PMC5636895; doi:10.1038/s41598-017-12615-w)
Supplement: Supplementary file 1 — Supplementary information [file 41598_2017_12615_MOESM1_ESM.pdf]

Supplementary information to the paper

Specific Binding of the Platelet Integrin αIIbβ3 to Fibrin Clots: A Potential Target to Destabilize Thrombi

Peter Höök, Rustem I. Litvinov, Oleg V. Kim, Shixin Xu, Zhiliang Xu, Joel S. Bennett, Mark S. Alber,

John W. Weisel

A model for forced unbinding of ligand-receptor complexes (LR)

Let P_1 and P_2 be the survival probabilities of states LR_1 and LR_2. The dynamic of the probabilities can then be calculated by the following system [1],

$$\frac{dP_1}{dt} = -(r_{12} + k_1)P_1 + r_{21}P_2, \quad (1)$$

$$\frac{dP_2}{dt} = -(r_{21} + k_2)P_2 + r_{12}P_1, \quad (2)$$

With initial conditions $P_1(t=0) = P_{1,0}$ and $P_2(t=0) = P_{2,0}$ where $P_{1,0} + P_{2,0} = 1$.

Here k_1 is the forced unbinding rate of the low-affinity complexes and is approximated by the Bell model: $k_1 = k_{10}\exp\left(\frac{fx_1}{K_BT}\right)$ with k_{10} being the force-free unbinding rate and x_1 being the critical elongation (or transition state distance) of the bond LR_1. k_2 is the forced unbinding rate of the high-affinity complexes and is approximated by $k_2 = k_{20}\exp\left(\frac{fx_2}{K_BT}\right)$, with k_{20} being the force-free unbinding rate and x_2 being the critical elongation (or transition state distance) of the bond LR_2. r_{12} and r_{21} are the transition rates from LR_1 to LR_2 and vice versa. $r_{12} = r_{12}^0\exp\left(-\frac{F_{12}}{K_BT}\right)$, $r_{21} = r_{21}^0\exp\left(-\frac{F_{21}}{k_BT}\right)$, where r_{12}^0 and r_{21}^0 are attempt frequencies, and F_{12} and F_{21} are free energy barrier height [1].

If we assume that the interconversion between the states is fast, i.e. $r_{12}P_1 = r_{21}P_2$ [2], then we define Ψ_0 as the force-free equilibrium constant,

$$\frac{P_1}{P_2} = \frac{P_{1,0}}{P_{2,0}} = \frac{r_{21}}{r_{12}} = \frac{r_{21}^0}{r_{21}^0}\exp\left(\frac{F}{K_BT}\right) = \Psi_0 \quad (3)$$

where $F = F_{12} - F_{21}$.

Then the similar Bell model is used for forced status,

$$\frac{P_1}{P_2} = \Psi_0 \exp\left(-\frac{f y_{12}}{K_B T}\right), \quad (4)$$

Where y_{12} is the distance between the energy wells LR_1 and LR_2.

By adding (1) and (2) , we could obtain the following equation,

$$\frac{dP_1}{dt} + \frac{dP_2}{dt} = -k_1 P_1 - k_2 P_2 = -\left(k_1 \Psi_0 \exp\left(-\frac{f y_{12}}{K_B T}\right) + k_2\right) P_2 = -\frac{k_1 \Psi_0 + k_2 \exp\left(\frac{f y_{12}}{K_B T}\right)}{\exp\left(\frac{f y_{12}}{K_B T}\right)} P_2. \quad (5)$$

At the same time, we have

$$\frac{P_1 + P_2}{\exp\left(\frac{f y_{12}}{K_B T}\right) + \Psi_0} = \frac{\left(1 + \Psi_0 \exp\left(-\frac{f y_{12}}{K_B T}\right)\right) P_2}{\exp\left(\frac{f y_{12}}{K_B T}\right) + \Psi_0} = \frac{P_2}{\exp\left(\frac{f y_{12}}{K_B T}\right)} . \quad (6)$$

Combining eqns (5-6) and denoting $P = P_1 + P_2$, yields

$$\frac{dP(t)}{dt} = -\frac{\left(k_1 \Psi_0 + k_2 \exp\left(\frac{f y_{12}}{K_B T}\right)\right) P(t)}{\Psi_0 + \exp\left(\frac{f y_{12}}{K_{BT}}\right)}, \quad (7)$$

$$P(t = 0) = 1. \quad (8)$$

[1] V. Barsegov and D. Thirumalai, "Dynamics of unbinding of cell adhesion molecules: Transition from catch to slip bonds," *Proc. Natl. Acad. Sci. USA.,* vol. 102, no. 6, pp. 1835-1839, 2005.

[2] E. Evans, A. Leung, V. Heinrich, and C. Zhu, "Mechanical switching and coupling between two dissociation pathways in a P-selectin adhesion bond," *Proc. Natl. Acad. Sci. USA,* vol. 101, pp. 11281-11286, 2004.
